# Supplementary figures and images for: The efficacy of Lacticaseibacillus paracasei MSMC39-1 and Bifidobacterium animalis TA-1 probiotics in modulating gut microbiota and reducing the risk of the characteristics of metabolic syndrome: A randomized, double-blinded, placebo-controlled study
Source: PLoS One. 2025 Jan 10;20(1):e0317202. doi: 10.1371/journal.pone.0317202 (PMC11723615; doi:10.1371/journal.pone.0317202)

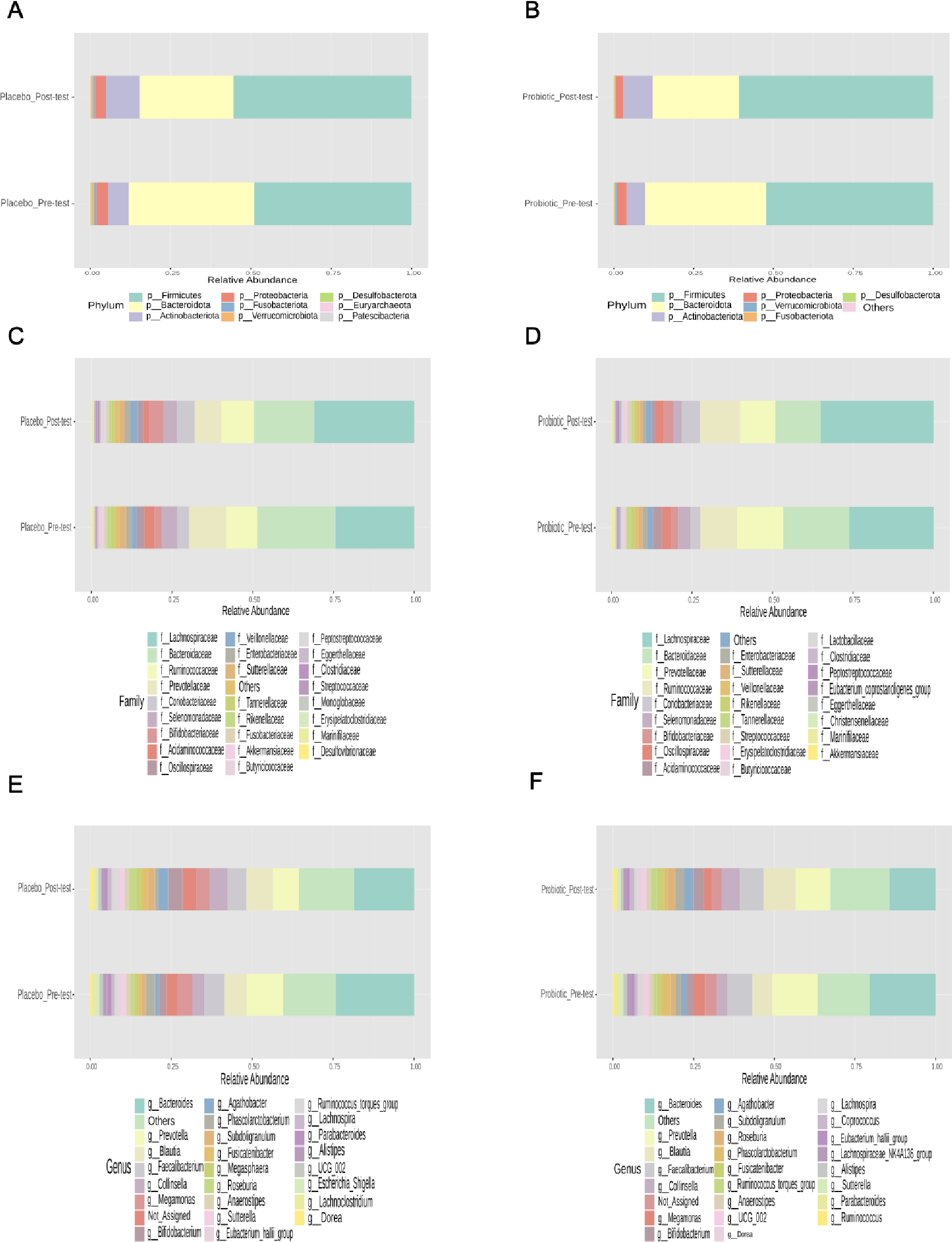

Supplement: S1 Fig — Relative abundance in the placebo and probiotics groups, pre-intervention and post-intervention, at the phylum (A and B), family (C and D), and genus (E and F) levels, respectively. (TIF) [file pone.0317202.s001.tif]
