# Supplementary material for: The efficacy of Lacticaseibacillus paracasei MSMC39-1 and Bifidobacterium animalis TA-1 probiotics in modulating gut microbiota and reducing the risk of the characteristics of metabolic syndrome: A randomized, double-blinded, placebo-controlled study
Source: PLoS One. 2025 Jan 10;20(1):e0317202. doi: 10.1371/journal.pone.0317202 (PMC11723615; doi:10.1371/journal.pone.0317202)
Supplement: S1 Table — (DOCX) [file pone.0317202.s002.docx]

**S1 Table.** **Baseline characteristics of the intervention and control groups.**

| **Variables** | **Placebo**  **(n = 27)** | **Probiotics**  **(n = 31)** | **P-value** |
| --- | --- | --- | --- |
| Males/ Females [n (%] | 8/19 (29.63/70.37) | 9/22 (29.03/70.97) | 0.960^3^ |
| Age (years) | 43.89 ± 7.54 | 42.29 ± 7.39 | 0.419^1^ |
| Weight (kg) | 75.90 (63.50, 79.10) | 69.40 (61.80, 91.00) | 0.944^2^ |
| BMI (kg/m^2^) | 28.12 ± 5.63 | 28.27 ± 6.65 | 0.930^1^ |
| Waist circumference (cm) | 34.50 (32.50, 38.00) | 34.50 (30.00, 39.00) | 0.708^2^ |
| Hip circumference (cm) | 41.81 ± 4.55 | 41.70 ± 4.85 | 0.929^1^ |
| SBP (mmHg) | 120 (110, 127) | 127 (115, 137) | 0.041^2^ |
| DBP (mmHg) | 80 (73, 86) | 82 (72, 87) | 0.932^2^ |
| Total cholesterol (mg/dl) | 239.11 ± 29.85 | 238.61 ± 30.73 | 0.951^1^ |
| Triglyceride (mg/dl) | 126.78 ± 51.43 | 127.84 ± 55.57 | 0.940^1^ |
| HDL-C (mg/dl) | 54 (47, 74) | 53 (45, 70) | 0.493^2^ |
| LDL-C (mg/dl) | 166 (147, 186) | 171 (146, 188) | 0.821^2^ |
| FBG (mg/dl) | 92.67 ± 11.35 | 93.77 ± 10.04 | 0.695^1^ |
| HbA1c (mg%) | 5.59 ± 0.39 | 5.63 ± 0.41 | 0.659^1^ |
| Creatinine (mg/dl) | 0.71 (0.62, 0.89) | 0.70 (0.66, 0.83) | 0.925^2^ |
| eGRF (ml/min/1.73^2^) | 107.73 (89.53, 111.57) | 105.53 (93.55, 112.19) | 0.703^2^ |
| AST (IU/L) | 19 (16, 22) | 19 (16, 26) | 0.870^2^ |
| ALT (IU/L) | 16 (12, 24) | 18 (13, 27) | 0.563^2^ |

^1^ Independent t-test (mean ± SD); ^2^ Mann-Whitney U test (median [interquartile range]); ^3^ Chi-square test; ALT, alanine aminotransferase; AST, aspartate aminotransferase; BMI, body mass index; DBP, diastolic blood pressure; FBG, fasting blood glucose; HbA1c, hemoglobin A1c; HDL-C, high-density lipoprotein cholesterol; IU/L, international units per liter; kg, kilogram; LDL-C, low-density lipoprotein cholesterol; mmHg, millimeters of mercury; mg, milligrams; mg/dl, milligrams per deciliter; SBP, systolic blood pressure
