# Supplementary material for: The efficacy of Lacticaseibacillus paracasei MSMC39-1 and Bifidobacterium animalis TA-1 probiotics in modulating gut microbiota and reducing the risk of the characteristics of metabolic syndrome: A randomized, double-blinded, placebo-controlled study
Source: PLoS One. 2025 Jan 10;20(1):e0317202. doi: 10.1371/journal.pone.0317202 (PMC11723615; doi:10.1371/journal.pone.0317202)
Supplement: S2 Table — (DOCX) [file pone.0317202.s003.docx]

**S2 Table.** **Clinical and laboratory characteristics of male participants.**

| **Variables** | **Placebo**  **(n = 8)** | **Probiotics**  **(n = 9)** | **P-value** |
| --- | --- | --- | --- |
| Weight (kg) | 0.55 (-1.6, 1.45) | -3.30 (-4.40, -3.00) | 0.004^2^ |
| BMI (kg/m^2^) | 0.18 (-0.53, 0.52) | 1.23 (1.36, 1.04) | 0.004^2^ |
| Waist circumference (cm) | -0.50 (-1.50, 1.25) | -2.00 (-3.00, -1.50) | 0.063^2^ |
| Hip circumference (cm) | -0.48 ± 0.63 | -2.53 ± 0.80 | <0.0011 |
| SBP (mmHg) | -1.00 ± 13.10 | -16.33 ± 15.70 | 0.047^1^ |
| DBP (mmHg) | -3.38 ± 4.84 | -6.00 ± 6.42 | 0.361^1^ |
| Total cholesterol (mg/dl) | -4.00 (-17.00, 20.00) | -37.50 (-59.00, -18.00) | 0.075^2^ |
| Triglyceride (mg/dl) | 3.00 (-9.00, 39.00) | -2.50 (-30.00, 1.00) | 0.038^2^ |
| HDL-C (mg/dl) | -0.25 ± 8.01 | 4.33 ± 5.05 | 0.110^1^ |
| LDL-C (mg/dl) | -10.88 ± 21.47 | -31.11 ± 38.32 | 0.207^1^ |
| FBG (mg/dl) | 1.00 (-12.5, 2.50) | 3.00 (-6.00 -, 10.00) | 0.288^2^ |
| HbA1c (mg%) | -0.01 ± 0.20 | -0.12 ± 0.20 | 0.278^1^ |
| Creatinine (mg/dl) | 0.04 ± 0.19 | 0.07 ± 0.14 | 0.720^1^ |
| eGRF (ml/min/1.73^2^) | -5.08 ± 13.86 | -3.37 ± 13.78 | 0.803^1^ |
| AST (IU/L) | 1.00 (-2.50, 5.50) | 2.00 (-1.00, 3.00) | 0.595^2^ |
| ALT (IU/L) | 2.88 ± 10.53 | 7.56 ± 15.21 | 0.478^1^ |

^1^ Independent t-test (mean ± SD); ^2^ Mann-Whitney U test (median [interquartile range]); ALT, alanine aminotransferase; AST, aspartate aminotransferase; BMI, body mass index; DBP, diastolic blood pressure; FBG, fasting blood glucose; HbA1c, hemoglobin A1c; HDL-C, high-density lipoprotein cholesterol; IU/L, international units per liter; kg, kilogram; LDL-C, low-density lipoprotein cholesterol; mmHg, millimeters of mercury; mg, milligrams; mg/dl, milligrams per deciliter; SBP, systolic blood pressure
