# Supplementary material for: The efficacy of Lacticaseibacillus paracasei MSMC39-1 and Bifidobacterium animalis TA-1 probiotics in modulating gut microbiota and reducing the risk of the characteristics of metabolic syndrome: A randomized, double-blinded, placebo-controlled study
Source: PLoS One. 2025 Jan 10;20(1):e0317202. doi: 10.1371/journal.pone.0317202 (PMC11723615; doi:10.1371/journal.pone.0317202)
Supplement: S3 Table — (DOCX) [file pone.0317202.s004.docx]

**S3 Table.** **Clinical and laboratory characteristics of female participants.**

| **Variables** | **Placebo**  **(n = 19)** | **Probiotics**  **(n = 12)** | **P-value** |
| --- | --- | --- | --- |
| Weight (kg) | -1.10 (-2.70, 2.00) | 3.95 (-4.70, 2.90) | <0.001^2^ |
| BMI (kg/m^2^) | -0.38 ± 0.65 | -1.52 ± 0.82 | <0.001^1^ |
| Waist circumference (cm) | -0.97 ± 1.64 | -2.55 ± 1.01 | <0.001^1^ |
| Hip circumference (cm) | -0.66 ± 0.62 | -2.07 ± 0.98 | <0.001^1^ |
| SBP (mmHg) | -1.00 (-7.00, 6.00) | -9.00 (-13.00, -3.00) | 0.008^2^ |
| DBP (mmHg) | -6.00 (-11.00, 1.00) | 3.50 (-13.00, 2.00) | 0.773^2^ |
| Total cholesterol (mg/dl) | -2.05 ± 26.98 | -38.95 ± 22.87 | <0.001^1^ |
| Triglyceride (mg/dl) | 8.32 ± 46.00 | -11.64 ± 23.21 | 0.081^1^ |
| HDL-C (mg/dl) | 0.00 (-4.00, 4.00) | 3.00 (1.00, 7.00) | 0.037^2^ |
| LDL-C (mg/dl) | 2.47 ± 22.34 | -43.59 ± 20.54 | <0.001^1^ |
| FBG (mg/dl) | -2.74 ± 9.53 | -3.00 ± 11.72 | 0.938^1^ |
| HbA1c (mg%) | -0.06 ± 0.21 | -0.15 ± 0.24 | 0.200^1^ |
| Creatinine (mg/dl) | 0.08 (0.01, 0.11) | 0.06 (0.00, 0.13) | 0.666^2^ |
| eGRF (ml/min/1.73^2^) | -7.09 ± 12.46 | -7.63 ± 10.15 | 0.803^1^ |
| AST (IU/L) | 2.00 (-3.00, 5.00) | 0.00 (-5.00, 2.00) | 0.088^2^ |
| ALT (IU/L) | -1.00 (-3.00, 6.00) | 0.00 (-4.00, 3.00) | 0.937^2^ |

^1^ Independent t-test (mean ± SD); ^2^ Mann-Whitney U test (median [interquartile range]); ALT, alanine aminotransferase; AST, aspartate aminotransferase; BMI, body mass index; DBP, diastolic blood pressure; FBG, fasting blood glucose; HbA1c, hemoglobin A1c; HDL-C, high-density lipoprotein cholesterol; IU/L, international units per liter; kg, kilogram; LDL-C, low-density lipoprotein cholesterol; mmHg, millimeters of mercury; mg, milligrams; mg/dl, milligrams per deciliter; SBP, systolic blood pressure
