# Supplementary material for: The efficacy of Lacticaseibacillus paracasei MSMC39-1 and Bifidobacterium animalis TA-1 probiotics in modulating gut microbiota and reducing the risk of the characteristics of metabolic syndrome: A randomized, double-blinded, placebo-controlled study
Source: PLoS One. 2025 Jan 10;20(1):e0317202. doi: 10.1371/journal.pone.0317202 (PMC11723615; doi:10.1371/journal.pone.0317202)
Supplement: S4 Table — (DOCX) [file pone.0317202.s005.docx]

**S4 Table. Multivariate regression analysis of clinical and laboratory changes.**

| **Variables** | **Placebo**  **(n = 27)** | **Probiotics**  **(n = 31)** | **Coef. (95% CI)** | **P-value** |
| --- | --- | --- | --- | --- |
| Weight (kg) | -0.69 ± 1.91 | -4.09 ± 2.70 | -3.06 (-4.37, -1.74) | <0.001 |
| BMI (kg/m^2^) | -0.26 ± 0.71 | -1.52 ± 0.92 | -1.16 (-1.62, -0.69) | <0.001 |
| Waist circumference (cm) | -0.70 ± 1.73 | -2.12 ± 2.03 | -1.56 (-2.57, -5.55) | 0.003 |
| Hip circumference (cm) | -0.60 ± 0.62 | -2.21 ± 0.94 | -1.60 (-2.06, -1.14) | <0.001 |
| SBP (mmHg) | 0.00 ± 10.18 | -11.65 ± 13.77 | -6.74 (-11.28, -2.20) | 0.004 |
| DBP (mmHg) | -4.33 ± 8.23 | -5.35 ± 9.96 | 0.60 (-4.39, 5.60) | 0.810 |
| Total cholesterol (mg/dl) | -4.00 ± 27.87 | -38.84 ± 27.47 | -33.45 (-49.19, -17.71) | <0.001 |
| Triglyceride (mg/dl) | 2.96 ± 49.57 | -18.52 ± 26.54 | -18.93 (-40.11, 2.24) | 0.079 |
| HDL-C (mg/dl) | -0.89 ± 8.71 | 4.55 ± 6.54 | 5.81 (1.47, 10.16) | 0.010 |
| LDL-C (mg/dl) | -4.96 ± 22.02 | -39.97 ± 26.83 | -35.09 (-49.15, -21.03) | <0.001 |
| FBG (mg/dl) | -3.19 ± 10.03 | -0.87 ± 12.43 | 0.89 (-5.43, 7.20) | 0.780 |
| HbA1c (mg%) | -0.04 ± 0.20 | -0.14 ± 0.23 | -0.10 (-0.22, 0.16) | 0.087 |
| Creatinine (mg/dl) | 3.18 ± 16.20 | 0.07 ± 0.10 | -2.56 (-8.75, 3.62) | 0.410 |
| eGRF (ml/min/1.73^2^) | -6.50 ± 12.65 | -6.40 ± 11.25 | 0.10 (-6.65, 6.84) | 0.977 |
| AST (IU/L) | 3.22 ± 11.26 | 0.16 ± 7.14 | -3.20 (-8.44, 2.04) | 0.226 |
| ALT (IU/L) | 1.59 ± 9.68 | 3.03 ± 12.31 | 1.03 (-5.24, 7.29) | 0.744 |

Regression analysis (placebo as reference), adjusted for sex, age, and baseline systolic blood pressure. ALT, alanine aminotransferase; AST, aspartate aminotransferase; BMI, body mass index; DBP, diastolic blood pressure; FBG, fasting blood glucose; HbA1c, hemoglobin A1c; HDL-C, high-density lipoprotein cholesterol; IU/L, international units per liter; kg, kilogram, LDL-C, low-density lipoprotein cholesterol; mmHg, millimeters of mercury; mg, milligrams; mg/dl, milligrams per deciliter; SBP, systolic blood pressure
