# Supplementary material for: The efficacy of Lacticaseibacillus paracasei MSMC39-1 and Bifidobacterium animalis TA-1 probiotics in modulating gut microbiota and reducing the risk of the characteristics of metabolic syndrome: A randomized, double-blinded, placebo-controlled study
Source: PLoS One. 2025 Jan 10;20(1):e0317202. doi: 10.1371/journal.pone.0317202 (PMC11723615; doi:10.1371/journal.pone.0317202)
Supplement: S5 Table — (DOCX) [file pone.0317202.s006.docx]

**S5 Table. Quality of life assessment results.**

| **Assessment** | **Placebo**  **(n = 27)** | **Probiotics**  **(n = 31)** | **P-value** |
| --- | --- | --- | --- |
| Frequency of defecation per week (5 points) | 4.35 ± 0.59 | 4.75 ± 0.25 | 0.460^1^ |
| Difficulty in defecation (5 points) | 4.43 ± 0.57 | 4.50 ± 0.50 | 0.421^1^ |
| Stomach pain or abdominal discomfort (5 points) | 4.39 ± 0.58 | 4.45 ± 0.55 | 0.695^1^ |
| Abdominal distension or bloating (5 points) | 4.20 ± 0.73 | 4.36 ± 0.64 | 0.483^1^ |
| Duration of defecation per time (5 points) | 4.34 ± 0.46 | 4.53 ± 0.47 | 0.951^1^ |
| Use of laxatives and/or enemas (5 points) | 4.36 ± 0.48 | 5.00 ± 0.00 | 0.659^1^ |
| Overall satisfaction (5 points) | 4.38 ± 0.48 | 4.58 ± 0.42 | 0.936^1^ |

^1^ Independent t-test (mean ± SD)
