# Supplementary material for: The efficacy of Lacticaseibacillus paracasei MSMC39-1 and Bifidobacterium animalis TA-1 probiotics in modulating gut microbiota and reducing the risk of the characteristics of metabolic syndrome: A randomized, double-blinded, placebo-controlled study
Source: PLoS One. 2025 Jan 10;20(1):e0317202. doi: 10.1371/journal.pone.0317202 (PMC11723615; doi:10.1371/journal.pone.0317202)
Supplement: Attachment — Submitted filename: Responses to review 2.docx [file pone.0317202.s007.docx]

**Response to Reviewer 2 Comments**

Title: Efficacy of *Lacticaseibacillus paracasei* MSMC39-1 and *Bifidobacterium animalis* TA-1 probiotics to modulate gut microbiota and reduce the risk of characteristics of metabolic syndrome: A randomized, double-blinded, placebo-controlled study

The authors would like to express our sincere gratitude for your valuable suggestions regarding the title, introduction, data presentation, clarification of the regimen, and overall understanding of this study. Your expertise has greatly contributed to refining and enhancing our manuscript. Moreover, your guidance has been instrumental in our growth and development as researchers and writers. Thank you once again for your insightful feedback and support.

We have carefully considered all your comments and have made the necessary revisions to address the issues you highlighted as follows.

**Comments from reviewer**

**Comment 1:** However, the writing style requires a more professional tone. I strongly recommend a thorough review by a native English speaker.

**Response 1:** The English language throughout the manuscript has been revised and corrected in accordance with the reviewer's suggestions.

**Comment 2:** Title: Instead of “reduce risk factors of metabolic syndrome” it can be “reduce the risk of characteristics of metabolic syndrome”

**Response 2:** Thank you so much for your valuable suggestions. The authors have revised the title as follows.

“Efficacy of *Lacticaseibacillus paracasei* MSMC39-1 and *Bifidobacterium animalis* TA-1 probiotics to modulate gut microbiota and reduce the risk of characteristics of metabolic syndrome: A randomized, double-blinded, placebo-controlled study”

**Comment 3:** The introduction is difficult to follow: and needs more organization. Adding subheadings such as “The effect of probiotics on body weight and composition”, “The effect of probiotics on lipid metabolism”, “The effect of probiotics on glucose metabolism” etc.

**Response 3:** The authors have revised the introduction, adding subheadings before paragraph 4 and paragraph 5 on page 3 of the introduction, and edited the text, as follows;

“**The effect of probiotics on body weight and composition**

Probiotics have emerged as a potential strategy for managing body weight and metabolic syndrome by influencing the gut microbiome. They are commonly used to maintain or restore a healthy microbial balance. Certain probiotic strains may reduce the risk of metabolic syndrome by modulating inflammation. A meta-analysis has shown that, compared to a placebo, probiotics can significantly reduce body weight, fat mass, and body mass index (BMI), highlighting their role as dietary agents in improving metabolic health (13).

**The effect of probiotics on lipid metabolism and glucose metabolism**

Several probiotic species, including *Bifidobacterium longum*, *Lactobacillus acidophilus*, and *Lactobacillus gasseri*, have demonstrated beneficial effects, such as the enhancement of lipid metabolic pathways, upregulation of genes involved in carbohydrate transport and metabolism, and improved intestinal digestion. Additionally, these probiotics have been shown to modulate bile acid metabolism, reduce plasma glucose, cholesterol, lipoproteins, and triglycerides, promote bile salt deconjugation, and contribute to weight loss (14). In one mice study, after a 12-week intervention, Thiennimitr et al. assessed the effects of probiotic *L. paracasei* HII01 (10^8^ CFU/mL), prebiotic xylo-oligosaccharides (XOS) (10%), and synbiotic on the improvement of gut dysbiosis. Supplementation improved both dyslipidemia and insulin sensitivity in obese mice, leading to better overall metabolic function (15). A randomized placebo-controlled clinical trial studied *L. plantarum* PBS067, *L. acidophilus* PBS066, and *L. reuteri* PBS072 with prebiotics in patients with metabolic syndrome. The results showed they could significantly reduce glucose, lipid, and inflammatory mediators (16).**”**

**Comment 4:** On page 4: “The dyslipidemia and insulin sensitivity in obese mice improved equally when the supplements were given. This improved the animals' metabolic dysfunction”. Please explain the meaning of “: equally”

**Response 4:** The authors have removed “equally” and revised the text in paragraph 3 on page 3 to provide a clearer and more precise description of the study's results, as follows;

**“**Several probiotic species, including *Bifidobacterium longum*, *Lactobacillus acidophilus*, and *Lactobacillus gasseri*, have demonstrated beneficial effects, such as the enhancement of lipid metabolic pathways, upregulation of genes involved in carbohydrate transport and metabolism, and improved intestinal digestion. Additionally, these probiotics have been shown to modulate bile acid metabolism, reduce plasma glucose, cholesterol, lipoproteins, and triglycerides, promote bile salt deconjugation, and contribute to weight loss (14). In one mice study, after a 12-week intervention, Thiennimitr et al. assessed the effects of probiotic *L. paracasei* HII01 (10^8^ CFU/mL), prebiotic xylo-oligosaccharides (XOS) (10%), and synbiotic on the improvement of gut dysbiosis. Supplementation improved both dyslipidemia and insulin sensitivity in obese mice, leading to better overall metabolic function (15). A randomized placebo-controlled clinical trial studied *L. plantarum* PBS067, *L. acidophilus* PBS066, and *L. reuteri* PBS072 with prebiotics in patients with metabolic syndrome. The results showed they could significantly reduce glucose, lipid, and inflammatory mediators (16).”

**Comment 5:** Study Design: Please justify including both genders in this study. Considering the difference in metabolism between males and females on one hand and females in peri- vs. post-menopausal status (age range 18-60), makes it difficult to interpret the results.

Suggestion: Include a separate analysis for males and females in addition to the current analysis.

**Response 5:** The authors have incorporated Supplementary Tables S2 and S3 and revised the first paragraph on page 9 to include the relevant text.

**Table S2.** Clinical and laboratory characteristics of male participants in the probiotics and placebo groups (N=58)

**Table S3.** Clinical and laboratory characteristics of female participants in the probiotics and placebo groups (N=58)

Following the intervention in the probiotics group, reductions were observed in total cholesterol, triglycerides, LDL-C, and HbA1c levels, along with an increase in HDL-C, when compared to the placebo group in both male and female participants (Tables S2-S3).

**Comment 6:** Study Design: In inclusion criteria, the citation (5) is not consistent with the way that the authors designed the inclusion/exclusion criteria. Please justify the current method utilized here.
Here is the methodology from citation 5: “MetS was defined by the NCEP ATP III-2005 criterion [15], that is, a person who has 3 or more of the following criteria: (1) elevated waist circumference (EWC): waist circumference ≥102 cm in men and ≥88 cm in women; (2) elevated blood pressure: blood pressure ≥130/85 mm Hg or drug treatment of previously diagnosed hypertension; (3) reduced HDL-C: <40 mg/dL in men and <50 mg/dL in women or specific treatment for reduced HDL-C; (4) elevated TGs: TG level ≥150 mg/dL or drug treatment for elevated TG; and (5) elevated fasting glucose: fasting glucose level of ≥100 mg/L or drug treatment for elevated glucose and previously diagnosed type 2 diabetes.”

**Response 6:** The authors greatly appreciate your thoughtful suggestion, and as a result, we have revised the inclusion criteria on page 4, as follows;

“**Inclusion criteria**

The inclusion criteria for participants in this study required individuals to meet the following conditions: (1) have an age range of 18 to 60 years, and (2) exhibit three or more of the following criteria: (a) elevated waist circumference (≥102 cm in men and ≥88 cm in women), (b) elevated blood pressure (≥130/85 mm Hg), (c) reduced high-density lipoprotein cholesterol (HDL-C) levels (<40 mg/dL in men and <50 mg/dL in women), (d) elevated triglycerides (≥150 mg/dL), and (e) elevated fasting blood glucose (100-125 mg/dL).”

**Comment 7:** Study Design: The regiment for probiotics is not described: frequency, and timing (within or between meals) are unclear. What is the nature/contact of the placebo? Please justify the length of the study (12 weeks).

**Response 7:** The authors have revised and expanded the text in paragraph 1 on page 4 to provide a clearer description of the probiotic regimen and the nature of the placebo and justify the length of the study (12 weeks).

Study Design: paragraph 1 on page 4

**“**This study was a randomized, double-blind, controlled, parallel-design trial. The study population comprised 60 participants who met the inclusion criteria for metabolic syndrome risk factors and were recruited from the outpatient clinic at the Faculty of Medicine, Srinakharinwirot University, between September 29, 2022, and April 5, 2023. Stratified permuted block randomization was utilized to assign participants into two groups. Group 1 received probiotics, consisting of *Lacticaseibacillus paracasei* MSMC39-1 (formerly *Lactobacillus paracasei*) at a dose of 1.0 x 10^9^ colony-forming units (CFU) and *Bifidobacterium animalis* TA-1 (1.0 x 10^9^ CFU), along with a vegetable-based pellet. Group 2 received a placebo vegetable-based pellet. Participants in each group consumed five tablets (250 mg per tablet), totaling 1,250 milligrams daily, 60 minutes before breakfast, for 12 weeks.”

Discussion: paragraph 6 on page 10 and paragraph 1 on page 11

**“**This double-blind, randomized controlled trial investigates the efficacy of *Lacticaseibacillus paracasei* MSMC39-1 and *Bifidobacterium animalis* TA-1 in reducing metabolic syndrome risk factors, particularly LDL-C levels, a key risk factor for coronary artery disease and other cardiovascular conditions (20). Vegetable-based pellets were used as the placebo in both groups and had no effect on the study outcomes. The primary objective was to evaluate the effectiveness of the probiotics in reducing the risk of characteristics of metabolic syndrome.”

Discussion: paragraph 3 on pages 11

The current study conducted a 12-week follow-up to evaluate differences in laboratory results, in alignment with the findings from the previous study.

**Comment 8:** Results: Table 1 does not represent the results. It can be relocated to the end of the study design section.

**Response 8:** In accordance with your suggestion, the authors have moved Table 1 to Supplementary Table S1.

**Comment 9:** Results: The presentation of the results in Table 2 is incomplete and unclear. Please add columns to present the absolute values of the variables. Please add columns to present the baseline and Week 12 values for each group.

**Response 9:** The authors have undertaken a major revision of Table 2 and renamed it as Table 1, as detailed below**.**

**Table 1.** Clinical and laboratory characteristics of the probiotics and placebo groups at baseline and week 12 (N=58)

| **Variables** | **Placebo**  **(n = 27)** | | **P-value** | **Probiotics**  **(n = 31)** | | **P-value** |
| --- | --- | --- | --- | --- | --- | --- |
|  | **Baseline** | **Week 12** |  | **Baseline** | **Week 12** |  |
| **Weight (kg)** | 75.90 (63.5 – 79.1) | 73.5 (61.3 – 79.2) | 0.089^2^ | 76.47 ± 21.72 | 72.38 ± 21.23 | <0.001^1^ |
| **BMI (kg/m^2^)** | 27.07 (24.06 –30.60) | 27.02 (24.39 –30.18) | 0.070^2^ | 28.27 ± 6.65 | 26.74 ± 6.56 | <0.001^1^ |
| **Waist circumference (cm)** | 34.5 (32.5 – 38) | 34 (32 – 37.5) | 0.024^2^ | 34.5 (30 – 39) | 32 (28 – 36.5) | <0.001^1^ |
| **Hips circumference (cm)** | 40.5 (39.5 – 43.5) | 40.5 (38.5 – 43.5) | <0.001^2^ | 41 (39 – 44) | 39 (37 – 42) | <0.001^1^ |
| **SBP (mmHg)** | 120 (110 – 127) | 119 (110 – 127) | 0.826^2^ | 127 (115 –137) | 115 (108 – 124) | <0.001^2^ |
| **DBP (mmHg)** | 80 (73 – 86) | 74 (70 – 80) | 0.002^2^ | 82 (72 – 87) | 77 (69 – 82) | 0.007^2^ |
| **Total cholesterol (mg/dl)** | 239.11 ± 29.85 | 235.11 ± 30.49 | 0.463^1^ | 242 (218 – 262) | 194 (186 – 219) | <0.001^2^ |
| **Triglyceride (mg/dl)** | 113 (84 – 168) | 107 (80 – 161) | 0.421^2^ | 127.84 ± 55.57 | 109.32 ± 41.85 | 0.001^1^ |
| **HDL-C (mg/dl)** | 54 (47 – 74) | 55 (47 – 69) | 0.791^2^ | 56.65 ± 14.37 | 61.19 ± 16.05 | 0.001^1^ |
| **LDL-C (mg/dl)** | 168.63 ± 29.68 | 163.67 ± 27.31 | 0.252^1^ | 171 (146 – 188) | 124 (118 – 134) | <0.001^2^ |
| **FBG (mg/dl)** | 92.67 ± 11.35 | 89.48 ± 9.20 | 0.111^1^ | 92 (87 -99) | 89 (83 – 98) | 0.543^2^ |
| **HbA1c (%)** | 5.59 ± 0.39 | 5.54 ± 0.35 | 0.260^1^ | 5.63 ± 0.41 | 5.49 ± 0.40 | 0.002^1^ |
| **Creatinine (mg/dl)** | 0.71 (0.62 – 0.89) | 0.79 (0.67 – 1.04) | 0.008^2^ | 0.75 ± 0.14 | 0.81 ± 0.14 | 0.001^1^ |
| **eGRF (ml/min/1.73^2^)** | 107.73 (89.53 – 111.57) | 96.78 (79.05 –105.53) | 0.009^2^ | 103.42 ±12.35 | 97.02 ± 14.23 | 0.004^1^ |
| **AST (IU/L)** | 19 (16 – 22) | 21 (17 – 24) | 0.129^2^ | 19 (16 – 26) | 18 (17 – 26) | 0.998^2^ |
| **ALT (IU/L)** | 16 (12 – 24) | 19 (11 – 28) | 0.524^2^ | 18 (13 – 27) | 16 (14 – 25) | 0.399^2^ |

^1^ Paired t-test, ^2^ Wilcoxon signed rank test, ALT, alanine aminotransferase; AST, aspartate aminotransferase; BMI, body mass index; DBP, diastolic blood pressure; FBG, fasting blood glucose; HbA1C, hemoglobin A1C; HDL-C, high-density lipoprotein cholesterol; IU/L, international units per liter; kg, kilogram, LDL-C, low-density lipoprotein cholesterol; mmHg, millimeters of mercury; mg/dl, milligrams per deciliter, ng/L, SBP, systolic blood pressure,

**Comment 10:** Results: The direction of current values as change is confusing. Please use negative for the reduction and positive for the increase. Current data for example shows a reduction in LDL in the placebo group assumingly compared with the probiotic group and a reduction in HDL in the probiotic group compared with the placebo group which is not consistent with the text.

**Response 10:** The authors have made substantial revisions to Table 2, as detailed below.

**Table 2.** The changes in 12 weeks of the intervention and control groups (N=58)

| **Variables** | **Placebo**  **(n = 27)** | **Probiotics**  **(n = 31)** | **P-value** |
| --- | --- | --- | --- |
| **Weight (kg)** | -0.90 (-2.00, 1.10) | -3.90 (-4.70, 3.00) | <0.001^2^ |
| **BMI (kg/m^2^)** | -0.37 (-0.65, 0.36) | -1.36 (-1.75, 1.07) | <0.001^2^ |
| **Waist circumference (cm)** | -0.50 (-2.00, 0.00) | -2.00 (-3.00, -2.00) | <0.001^2^ |
| **Hips circumference (cm)** | -0.60 ± 0.62 | -2.21 ± 0.94 | <0.001^1^ |
| **SBP (mmHg)** | -1 (-8, 7) | -10 (-24, -3) | 0.003^2^ |
| **DBP (mmHg)** | -5 (-9, 1) | -5 (-13, 1) | 0.913^2^ |
| **Total cholesterol (mg/dl)** | -4.00 ± 27.87 | -38.84 ± 27.47 | <0.001^1^ |
| **Triglyceride (mg/dl)** | 4 (-9, 33) | -9 (-43, 0) | 0.011^2^ |
| **HDL-C (mg/dl)** | 0 (-5, 4) | 4 (1, 7) | 0.007^2^ |
| **LDL-C (mg/dl)** | -4.96 ± 22.02 | -39.97 ± 26.83 | <0.001^1^ |
| **FBG (mg/dl)** | -3.19 ± 10.03 | -0.87 ± 12.43 | 0.443^1^ |
| **HbA1c (%)** | 0.00 (0.20, 0.10) | -0.10 (-0.20, 0.00) | 0.086^2^ |
| **Creatinine (mg/dl)** | 0.08 (-0.01, 0.11) | 0.05 (0.00, 0.13) | 0.981^2^ |
| **eGRF (ml/min/1.73^2^)** | -6.50 ± 12.65 | -6.40 ± 11.25 | 0.975^1^ |
| **AST (IU/L)** | 2 (-3, 5) | 0 (-3, 3) | 0.250^2^ |
| **ALT (IU/L)** | 0 (-3, 6) | 1 (-4, 5) | 0.827^2^ |

^1^ Independent t-test (mean ± SD), ^2^ Mann-Whitney U test (median (IQR)), ALT, alanine aminotransferase; AST, aspartate aminotransferase; BMI, body mass index; DBP, diastolic blood pressure; FBG, fasting blood glucose; HbA1C, hemoglobin A1C; HDL-C, high-density lipoprotein cholesterol; IU/L, international units per liter; kg, kilogram, LDL-C, low-density lipoprotein cholesterol; mmHg, millimeters of mercury; mg/dl, milligrams per deciliter, ng/L, SBP, systolic blood pressure

Your feedback has been greatly appreciated and has played a crucial role in enhancing this work. We hope the revisions align with your expectations and look forward to the possibility of collaborating again in the future.
