# Supplementary material for: The efficacy of Lacticaseibacillus paracasei MSMC39-1 and Bifidobacterium animalis TA-1 probiotics in modulating gut microbiota and reducing the risk of the characteristics of metabolic syndrome: A randomized, double-blinded, placebo-controlled study
Source: PLoS One. 2025 Jan 10;20(1):e0317202. doi: 10.1371/journal.pone.0317202 (PMC11723615; doi:10.1371/journal.pone.0317202)
Supplement: Attachment — Submitted filename: Responses to reviewer3.docx [file pone.0317202.s008.docx]

**Response to Reviewer 3 Comments**

Title: The efficacy of *Lacticaseibacillus paracasei* MSMC39-1 and *Bifidobacterium animalis* TA-1 probiotics in modulating gut microbiota and reducing the risk of the characteristics of metabolic syndrome: A randomized, double-blinded, placebo-controlled study

We are especially grateful for your valuable suggestions on the methodology, clarification of the statistical analysis, and insightful knowledge regarding this study. Your expertise has greatly contributed to the refinement of our manuscript and has played a pivotal role in our growth as researchers and writers. Your guidance has been instrumental in enhancing the quality of this work.

We have thoroughly reviewed all of your comments and have implemented the necessary revisions to address the concerns you raised, as detailed below.

**Comments from reviewer**

**Comment 1:**

The introduction should be shortened and made more adherent to the clinical potential impact of these findings. Subheadings should be removed

**Response 1:**

The introduction has been revised to focus more concisely on the clinical potential impact of the findings, emphasizing their relevance to patient outcomes and therapeutic implications. The unnecessary details have been removed to streamline the narrative and align it with the study's clinical significance. Additionally, all subheadings have been eliminated to ensure a smoother and more cohesive flow throughout the manuscript, as per your request. The authors have revised the second to fourth paragraphs of the introduction on pages 2-4 (lines 56-87) to include the relevant text as follows.

“The gastrointestinal tract is colonized by the intestinal microbiota, a key environmental factor that directly influences host health and contributes to the exacerbation of various diseases (6). Dysbiosis, an imbalance in the microbiota, reduces its diversity and function, leading to metabolic issues. A high-fat diet can worsen dysbiosis by increasing circulating lipopolysaccharide (LPS) and lipid levels, as well as compromising the intestinal barrier. Additionally, dysbiosis may contribute to chronic inflammation and the development of obesity, diabetes mellitus, and metabolic syndrome through interactions between genetic and environmental factors (7,8). Treatment for metabolic syndrome and obesity-related metabolic endotoxemia involves preventing microbial dysbiosis and maintaining intestinal barrier integrity (9).

According to Hill et al. (10), the consensus statement that the International Scientific Association for Probiotics and Prebiotics (ISAPP) proposed for the proper usage of the term probiotic refers to live microorganisms that, when administered in adequate amounts, confer a health benefit on the host. Commercial strains are more likely to contain probiotics from the genera *Lactobacillus* and *Bifidobacterium* (11). The key health benefits of probiotics include immune modulation, enhanced mineral and vitamin absorption, constipation relief, microbiome regulation, post-antibiotic microbiome stabilization, increased gastrointestinal resistance to pathogens, and pathogen reduction through short-chain fatty acid (SCFA) production (12).

Probiotics offer a potential strategy for managing body weight as well as metabolic syndrome by modulating the gut microbiome. They help maintain microbial balance and may reduce the risk of metabolic syndrome by controlling inflammation. A meta-analysis showed that probiotics, compared to placebos, significantly reduce body weight, fat mass, and the body mass index (BMI), supporting their role in improving metabolic health (13). Probiotic species like *Bifidobacterium longum*, *Lactobacillus acidophilus*, and *Lactobacillus gasseri* have demonstrated benefits by enhancing lipid metabolism, upregulating carbohydrate transport and metabolism genes, and improving digestion. They also modulate bile acid metabolism, reduce plasma glucose, cholesterol, and triglycerides, promote bile salt deconjugation, and aid in weight loss (14). A 12-week mice study confirmed that *L. paracasei* HII01, xylo-oligosaccharides (XOS), and synbiotics improved dyslipidemia and insulin sensitivity, enhancing metabolic functions (15). Similarly, a randomized placebo-controlled trial found *L. plantarum* PBS067, *L. acidophilus* PBS066, and *L. reuteri* PBS072, along with prebiotics significantly reduced glucose, lipids, and inflammatory mediators in metabolic syndrome patients (16).”

**Comment 2:** Methods: it should be added how the placebo was created
**Response 2:**

In response to the feedback, the Methods section has been updated to include details on the creation of the placebo. The authors have revised the first paragraphs of the Methods section on pages 3-4 (lines 105-109) to include the relevant text as follows.

“The tablets were manufactured at a good manufacturing practice (GMP) certified food production facility. Probiotics with a vegetable-based pellet and a placebo vegetable-based pellet were compressed into tablets using microcrystalline cellulose (MCC) as a binder to enhance cohesion. The tablets were coated with hydroxypropyl methylcellulose (HPMC) to protect against moisture.”

**Comment 3:** Methods: sample size calculation is totally not clear to me, and usually should be performed on primary outcomes

**Response 3:**

Thank you for your feedback. The sample size calculation has been revised for clarity and is now based on the primary outcome of the study. The authors have revised the first paragraphs of the Methods section, sample size calculation on page 5 (lines 162-168), as follows.

“The primary outcome was utilized to determine the sample size, based on previous research that investigated LDL-C changes between placebo and intervention groups (17). The placebo group exhibited a change of 0.17 ± 0.46, while the intervention group showed a difference of -0.40 ± 0.70. Using these values, the sample size was calculated with STATA software, setting α = 0.05 and power (1-β) = 0.90. The required sample size was determined to be 24 participants per group. To account for potential missing data (approximately 20%), the sample size was adjusted to approximately 28 participants per group.”

**Comment 4:** Methods: to correct for reduced sample size sometimes multivariate analysis is exploited. did authors exploit it?

**Response 4:**

In response to the query, multivariate regression analysis was employed to account for the reduced sample size and control for potential confounding factors. The multivariate regression analysis is presented in Table S4 and has been further explained in the Results section on page 9 (lines 279-285) to provide a detailed interpretation of the findings, as follows.

“Multivariate regression analysis revealed that the clinical and laboratory characteristics, as well as changes in variables between the probiotics and placebo groups, remained consistent. After 12 weeks of administration of the probiotic strains *L. paracasei* MSMC39-1 and *B. animalis* TA-1, a significant reduction in LDL-C after taking probiotics was achieved (the differences were 35.09, p-value <0.001). Additionally, participants in the probiotics group exhibited significant reductions in body weight, BMI, waist circumference, SBP, and total cholesterol compared to the placebo group at baseline and after 12 weeks (Table S4).”

Your feedback is sincerely appreciated and has been pivotal in improving this work. We look forward to the opportunity to collaborate again in the future and trust that the revisions align with your expectations.

Once again, we extend our gratitude for your valuable insights and for your support of the peer review process.
