# Supplementary material for: The efficacy of Lacticaseibacillus paracasei MSMC39-1 and Bifidobacterium animalis TA-1 probiotics in modulating gut microbiota and reducing the risk of the characteristics of metabolic syndrome: A randomized, double-blinded, placebo-controlled study
Source: PLoS One. 2025 Jan 10;20(1):e0317202. doi: 10.1371/journal.pone.0317202 (PMC11723615; doi:10.1371/journal.pone.0317202)
Supplement: Attachment — Submitted filename: Response to Reviewer 4.docx [file pone.0317202.s009.docx]

**Response to Reviewer 4 Comments**

Title: The efficacy of *Lacticaseibacillus paracasei* MSMC391 and *Bifidobacterium animalis* TA1 probiotics in modulating gut microbiota and reducing the risk of the characteristics of metabolic syndrome: A randomized, double-blinded, placebo-controlled study

We sincerely thank you for your thorough and constructive feedback on our manuscript. We deeply appreciate the time and effort dedicated to reviewing our work, and we have carefully considered each comment to revise and improve the manuscript accordingly. Below, we present a detailed, point-by-point response to the reviewers' comments. Revisions have been highlighted in the manuscript for ease of reference.

**Reviewer comments and our responses:**

1. Move the number of participants in the two groups to lines 37 and 38 of the abstract to avoid confusion.

**Response:** The number of participants in both groups has been moved to lines 37 and 38 in the abstract to enhance clarity, as follows.

“Fifty-eight participants with risk factors of metabolic syndrome according to the inclusion criteria were randomized into two groups and given probiotics (*Lacticaseibacillus paracasei* MSMC39-1 and *Bifidobacterium animalis* TA-1) (n=31) or a placebo (n=27).” (line 27)

2. Place the units of all parameters evaluated and detailed in the text. Example: line 45, the value 39.97 has no units. Revise these errors in the complete text.

**Response:** Units have been added to all parameters throughout the manuscript, including the example on line 45. The text has been thoroughly reviewed to ensure all numerical values are appropriately labeled, as follows.

- “The primary outcome was achieved by the probiotics group as their low-density lipoprotein-cholesterol level dramatically lowered compared to the placebo group (the difference was 39.97 ± 26.83 mg/dl, p-value <0.001).” (line 32)
- “Participants who met the following criteria were excluded: (1) they had been diagnosed with the following underlying diseases: diabetes mellitus (hemoglobin A1c [HbA1c] ≥ 6.5 mg%)” (line 147)
- “Following the administration of both strains of probiotics, namely *L. paracasei* MSMC39-1 and *B. animalis* TA-1 at 12 weeks, the difference in participants' body weight, BMI, and waist circumference, all significantly decreased from the baseline (3.90 kg, 1.36 kg/m^2^, and 2.00 cm, p-value <0.001 for body weight, BMI, and waist circumference, respectively), and the variation differed significantly from the placebo group (Table 2).” (line 256)
- “The total cholesterol, triglycerides, and LDL-C levels, considerably decreased after taking probiotics compared to before with statistical significance (the differences were 38.84 ± 27.47 mg/dl, p-value <0.001; 9 mg/dl, p-value 0.011; and 39.97 ± 26.83 mg/dl, p-value <0.001, respectively).” (line 262)
- “After 12 weeks of administration of the probiotic strains *L. paracasei* MSMC39-1 and *B. animalis* TA-1, a significant reduction in LDL-C after taking probiotics (39.97 ± 26.83 mg/dl) was achieved (p-value <0.001). Additionally, compared to the placebo group, participants in the probiotics group showed significant reductions at 12 weeks from baseline in body weight (4.09 ± 2.70 kg), BMI (1.52 ± 0.92 kg/m²), waist circumference (2.12 ± 2.03 cm), systolic blood pressure (11.65 ± 13.77 mmHg), and total cholesterol (38.84 ± 27.47 mg/dl) (Table S4).” (line 287-291)

3. Change the term percentage for its sign (%) in line 73 and revise these errors in the complete text.

**Response:** The term "percentage" has been replaced with the sign (%) in previous line 73 and similar instances throughout the manuscript.

4. Important statements are made in the introduction and in the discussions. These should be properly cited. There are long and important sentences that are supported with only one bibliographic reference. It is important to add more updated references.

**Response:** Additional updated references have been added to support important statements in both the introduction and discussion sections. These references are appropriately cited in the text, as follows.

- “This double-blind, randomized controlled trial investigated the efficacy of *Lacticaseibacillus paracasei* MSMC39-1 and *Bifidobacterium animalis* TA-1 in reducing metabolic syndrome risk factors, particularly LDL-C levels, which are a key risk factor for coronary artery disease and other cardiovascular conditions (33,49,50).” We added the reference numbers 33,49,50 (lines 348-351).
- “In this investigation, the LDL-C levels in the intervention group decreased significantly after receiving the probiotics compared to the placebo one. High levels of LDL-C are a risk factor for diabetes mellitus and cardiovascular disease (49,58–60).” We added the references number 49,58-60 (lines 372-374).
- “The breakdown of bile salts in the digestive tract is attributed to BSH, which is another way in which probiotics can decrease cholesterol. It is then eliminated through the feces and is in an insoluble state. Consequently, the liver must extract more cholesterol to produce bile salts. This is an indirect method of decreasing cholesterol (65,66).” We added the reference numbers 65,66 (lines 384-388).

5. Explain what technique was used to randomize the study population.

**Response:** Details of the randomization technique have been added to the methods section for clarification, as follows.

- “Stratified permuted block randomization was utilized to assign the participants into two groups.” (lines 98-99)

6. Put references in lines 176 to 178, 517 to 522, and other paragraphs such as these that do not have bibliographic references.

**Response:** Bibliographic references have been added to the mentioned lines and other relevant paragraphs to provide appropriate support for the statements made, as follows.

- “The probiotic strains used in this test can reduce sugar and lipid levels in both in vitro and in animal models (27–29).” References 27-29 have been added to support the information presented. (lines 120-121)
- “The breakdown of bile salts in the digestive tract is attributed to BSH, which is another way in which probiotics can decrease cholesterol. It is then eliminated through the feces and is in an insoluble state. Consequently, the liver must extract more cholesterol to produce bile salts. This is an indirect method of decreasing cholesterol (65,66).” References 65 and 66 have been added to support the information presented. (line 388)
- “The gastrointestinal tract is colonized by the intestinal microbiota, a key environmental factor that directly influences host health and contributes to the exacerbation of various diseases (6–9). Dysbiosis, an imbalance in the microbiota, reduces its diversity and function, leading to metabolic issues (10). A high-fat diet can worsen dysbiosis by increasing circulating lipopolysaccharide (LPS) and lipid levels, as well as compromising the intestinal barrier (11).” References 6-9, 10, and 11 have been added to support the information presented. (lines 57, 58, and 59)
- “The key health benefits of probiotics include immune modulation, enhanced mineral and vitamin absorption, constipation relief, microbiome regulation, post-antibiotic microbiome stabilization, increased gastrointestinal resistance to pathogens, and pathogen reduction through short-chain fatty acid (SCFA) production (17–19).” References 18 and 19 have been added to support the information presented. (line 72)
- “Low-density lipoprotein cholesterol (LDL-C) is considered a critical contributor to the development of cardiovascular disease (33).” Reference 33 has been added to support the information presented. (line 167)
- “The QIAamp DNA Stool Mini Kit (Qiagen, USA) was used to extract DNA (35). The quantity and quality of the DNA were assessed using nanodrop and electrophoresis (36). By using 2X KAPA hot-start ready mix and 515 F and 806R primers, the V4 hypervariable region of the 16S rRNA gene was amplified by polymerase chain reaction (PCR) (37). References 35 -37 have been added to support the information presented.” (lines 182-185)
- “After eight cycles of the previously described PCR condition, the 16S amplicons were purified using AMPure XP beads and indexed with a Nextera XT Index Kit (38). Ultimately, the PCR products underwent cleaning and pooling in preparation for the Illumina® MiSeq™250-base paired-end read sequencing and cluster formation (39,40).” References 38 -40 have been added to support the information presented.” (lines 189-191)
- “The classify-sklearn naive Bayes taxonomy classifier was adopted to assign taxonomy to amplicon sequence variants (ASVs) based on comparison with the Greengenes 13_8 99% operational taxonomic units (OTUs) reference sequences (42). By applying the Kruskal-Wallis test and a permutational multivariate analysis of variance (PERMANOVA) with 999 permutations, respectively, statistical tests of the alpha and beta diversity were carried out (43). A heat tree analysis was generated for pairwise comparisons of the taxonomic differences between microbial communities with the MicrobiomeAnalyst web-based platform (44). ” References 42 -44 have been added to support the information presented.” (lines 202-206)
- “Stata/SE 16.1 software (StataCorp LP, College Station, TX, USA) was employed to analyze the statistical data (47).” Reference 47 has been added to support the information presented. (line 218)
- “The Benjamini-Hochberg (FDR) correction was applied to p-values for multiple hypothesis testing (48).” Reference 48 has been added to support the information presented. (line 225)
- “In this investigation, the LDL-C levels in the intervention group decreased significantly after receiving the probiotics compared to the placebo one. High levels of LDL-C are a risk factor for diabetes mellitus and cardiovascular disease (49,58–60).” References 49,58–60 have been added to support the information presented. (line 374)
- “In this research, the total cholesterol and triglyceride levels decreased significantly after the consumption of the probiotics for 12 weeks. Compared with pre-intervention placebo values, total cholesterol declined by 16.05% in the probiotic group versus 1.67% in placebo, while triglycerides fell by 7.04% versus 3.54%, respectively. Although HDL-C decreased in the probiotic group, the change was not statistically significant. These results align with previous trials: one involving dyslipidemic patients showed a 13.6% reduction in total cholesterol in the *L. plantarum* group after 12 weeks (67), and another by Keleszade et al. found that six weeks of *L. plantarum* ECGC 13110402 use significantly lowered total cholesterol and LDL-C levels (68). Additionally, *B. longum* CCFM1077 reduced total cholesterol by 13.87% in hyperlipidemic patients, and synbiotic foods containing *L. sporogenes* and inulin improved triglycerides and HDL-C (69). Overall, both fermented milk products and probiotics significantly reduced total cholesterol and LDL-C, with interventions exceeding four weeks proving more effective (70).” This paragraph has been revised for conciseness while maintaining the original content and references 67-70 have been added to support the information presented. (lines 389-400)
- “Probiotics could influence SBP through several mechanisms related to gut microbiome modulation (71). These outcomes are a result of the complex interactions that occur between the host's physiological processes, their metabolic products, and the gut microbiome (72). Certain probiotic strains can ferment and create peptides that mimic the effects of angiotensin-converting enzyme inhibitors, a type of medication used to treat hypertension (73).” References 71-73 have been added to support the information presented. (lines 402-405)
- “These clinical trials have investigated the impact of probiotics on blood pressure, with some reporting modest reductions in SBP reductions (reduced SBP by 3.05 mmHg and DBP by 1.51 mmHg), which is consistent with our work (reduced SBP by 10 mmHg with statistical significance, DBP 5 mmHg with no statistical significance) (75,76).” References 75,76 have been added to support the information presented. (line 417)
- “In this study, the LDL-C levels showed a marked decrease in the probiotic group compared to the placebo group, dropping by 23.37% and 2.94%, respectively, following the intervention. These results are supported by a meta-analysis of randomized controlled trials, which reported that consuming probiotic *Lactobacillus* strains, particularly *L. reuteri* and *L. plantarum*, significantly reduced both total cholesterol and LDL-C levels (69). In a controlled, randomized, double-blind trial conducted among dyslipidemic patients, supplementation with a combination of three *L. plantarum* strains (CECT 7527, CECT 7528, and CECT 7529) resulted in a 17.6% reduction in LDL-C levels after 12 weeks compared to the placebo group (67). In another randomized trial, the administration of *Bifidobacterium longum* CCFM1077 to hyperlipidemic patients reduced LDL-C levels by approximately 13.88%. This benefit may be attributed to the proliferation of anti-obesity-related bacterial genera and favorable shifts in fecal metabolite profiles (77).” References 69, 67, and 77 have been added to support the information presented. (lines 419-430)
- “Moreover, higher quantities of SCFAs can be produced more effectively by a more diversified gut microbiota (78,79).” References 78,79 have been added to support the information presented. (line 436)
- “The fermentation of complex carbohydrates produces metabolites known as SCFAs (80,81). Although butyrate can only be generated by members of the phylum Firmicutes, members of the Bacteroidetes phylum can also create acetate (82,83). *Eubacterium rectale* and *Roseburia* show a positive connection with SCFAs (84).” References 80-84 have been added to support the information presented. (lines 438-440)
- “SCFAs can be produced by lactic acid bacteria, such as those of the genera *Lactobacillus* and *Bifidobacterium*, even though these are not classified under the SCFAs category (88).” Reference 88 has been added to support the information presented. (line 450)
- “This imbalance is caused by an overgrowth of opportunistic bacteria and can lead to various health issues, including inflammatory bowel disease (IBD) (90), obesity (91), and metabolic disorders (92).” References 90-92 have been added to support the information presented. (lines 458-459)
- “Patients with autoimmune disorders (96), type 2 diabetes (97), and atherosclerotic diseases (98) frequently have abnormally low levels of SCFA-producing gut microbes (99).” References 96-99 have been added to support the information presented. (lines 470-471)
- “Therefore, creating and introducing bacterial occupants (or co-inhabitants) that have the ability to cross-feed and increase/reduce the number of SCFA-producing bacteria in the gut could be a viable probiotic strategy to aid in the treatment of several human illnesses (78,79).” References 78-79 have been added to support the information presented. (line 484)
- “Patients with obesity typically have altered gut microbiota compositions, suggesting that the gut microbiome may be a contributing component to the development of obesity (103,104).” References 103-104 have been added to support the information presented. (line 486)
- “An increased risk of cardiovascular disease may be closely linked to diabetes mellitus (109). A changed gut microbiome can lead to oxidative stress-related diseases, as well as obesity, metabolic endotoxemia, B-cell dysfunction, and systemic inflammation (110,111).” References 109-111 have been added to support the information presented. (lines 502-503)
- “One of the key components of lipid metabolism is RNA transport. The tissue-specific targeting of RNA-based therapies is now possible, increasing the safety of this treatment (115,116). RNA-based therapies have the potential to dramatically improve outcomes for individuals who have or are at risk for atherosclerotic cardiovascular disease, and multiple putative target proteins have been identified (117). The biosynthesis of unsaturated fatty acids, which are considered healthy fats, can lower blood cholesterol levels and decrease the risks of cardiovascular diseases (118,119).” References 115-116, 118-119 have been added to support the information presented. (lines 525, 529)

7. Lines 183 to 185 are results; they should not be in methods.

**Response:** The content from lines 183 to 185 has been removed from the methods.

8. Justify why it was decided to evaluate the results at 12 weeks.

**Response:** The rationale for evaluating results at 12 weeks has been added to the discussion section, citing prior research highlighting 12 weeks as a sufficient timeframe to observe meaningful probiotic effects, as follows.

- “The gut microbiota typically requires weeks to months to exhibit measurable changes in response to probiotics. While short-term effects (e.g., changes in metabolite levels) can occur sooner, 12 weeks allows sufficient time for sustained microbial colonization, shifts in microbial diversity, and host responses (51,52). Many health benefits linked to probiotics, such as improved gastrointestinal function, immune response, or systemic inflammation, often manifest after 8–12 weeks (53–55). Therefore, evaluating at 12 weeks ensures that intermediate and longer-term outcomes can be captured reliably. A systematic review and meta-analysis of randomized clinical trials concluded that probiotic supplementation significantly reduces total cholesterol and LDL-C levels, with a stronger effect on LDL-C observed in interventions lasting longer than six weeks (56). Likewise, a randomized controlled study found that *L. plantarum* strains (CECT7527, CECT7528, and CECT7529) reduced LDL-C and total cholesterol after 12 weeks compared to placebo, with the LDL-C-lowering effect potentially influenced by individual gut microbiota differences (57). Similarly, this study conducted a 12-week follow-up to assess laboratory changes, aiming to validate and expand upon these findings. Furthermore, a 12-week intervention allows for observing effects while ensuring participant adherence. Longer durations may increase dropout rates, weakening the study. This timeframe helps reduce transient variations or short-term influences (e.g., dietary changes, seasonal effects) that could confound results.” (lines 355-371)

9. Improve the explanation of the sample size calculation. Why LDL-C is chosen for the calculation?

**Response:** The explanation for the sample size calculation has been expanded, including the rationale for selecting LDL-C as the primary parameter based on its clinical significance and prior studies. Elevated low-density lipoprotein cholesterol (LDL-C) is a key driver of cardiovascular disease, with the INTERHEART study identifying it as more critical than hypertension, diabetes, smoking, or obesity.

- “LDL-C is considered a critical contributor to the development of cardiovascular disease (6).” This paragraph has been added to emphasize the significance of LDL-C in the context of this study. (lines 166-167)

10. Make a table summarizing the results explained in lines 411 to 416.

**Response:** A summary table has been created to present the results from lines 411 to 416 more clearly in Table S5: Quality of Life Assessment Results Comparing the Probiotics and Placebo Groups (N=58), as follows.

- “To assess participants' tolerance and satisfaction with the intervention, a quality-of-life questionnaire was administered at the final visit (12 weeks). The seven items included: (1) frequency of defecation (5 points for daily, down to 1 point for none); (2) difficulty in defecation (5 points for never, down to 1 for always); (3) stomach pain (5 points for never, down to 1 for always); (4) bloating (5 points for never, down to 1 for always); (5) use of laxatives (5 for never, down to 1 for always); (6) duration of defecation (5 points for <5 minutes, down to 1 for >30 minutes); (7) overall satisfaction (5 points for strongly agree, down to 1 for strongly disagree). This evaluation provided insights into participants’ gastrointestinal health and satisfaction with the intervention.” We included this paragraph in the methods section to provide a detailed explanation of the quality-of-life assessment. (lines 125-132)
- We added Table S5: Quality of Life Assessment Results Comparing the Probiotics and Placebo Groups (N=58).

11. Correct Figure 4. Represent the placebo and postbiotic groups in the same color in A and B to avoid confusion. Improve the caption of the image. What exactly does A and B represent?

**Response:** Figure 4 has been updated to ensure consistency in the color representation of placebo and postbiotic groups in panels A and B. The caption has been revised to clearly explain what A and B represent.

12. In discussions, discuss how much cholesterol, triglycerides, glucose, etc., are reduced. Compare with the cited studies by expressing the percentages or values reported to know if the variability is similar or different from the study.

**Response:** The discussion has been expanded to include specific values and percentages for reductions in cholesterol, triglycerides, glucose, and other parameters. These values have been compared with the results of cited studies to highlight similarities and differences, as follows.

- “In this study, the LDL-C levels showed a marked decrease in the probiotic group compared to the placebo group, dropping by 23.37% and 2.94%, respectively, following the intervention. These results are supported by a meta-analysis of randomized controlled trials, which reported that consuming probiotic *Lactobacillus* strains, particularly *L. reuteri* and *L. plantarum*, significantly reduced both total cholesterol and LDL-C levels (69). In a controlled, randomized, double-blind trial conducted among dyslipidemic patients, supplementation with a combination of three *L. plantarum* strains (CECT 7527, CECT 7528, and CECT 7529) resulted in a 17.6% reduction in LDL-C levels after 12 weeks compared to the placebo group (67). In another randomized trial, the administration of *Bifidobacterium longum* CCFM1077 to hyperlipidemic patients reduced LDL-C levels by approximately 13.88%. This benefit may be attributed to the proliferation of anti-obesity-related bacterial genera and favorable shifts in fecal metabolite profiles (77).” We revised this paragraph to be more concise while retaining the details and included specific percentages for LDL-C reductions to facilitate comparison with the cited study. (lines 419-430)
- “In this research, the total cholesterol and triglyceride levels decreased significantly after the consumption of the probiotics for 12 weeks. Compared with pre-intervention placebo values, total cholesterol declined by 16.05% in the probiotic group versus 1.67% in placebo, while triglycerides fell by 7.04% versus 3.54%, respectively. Although HDL-C decreased in the probiotic group, the change was not statistically significant. These results align with previous trials: one involving dyslipidemic patients showed a 13.6% reduction in total cholesterol in the *L. plantarum* group after 12 weeks (67), and another by Keleszade et al. found that six weeks of *L. plantarum* ECGC 13110402 use significantly lowered total cholesterol and LDL-C levels (68). Additionally, *B. longum* CCFM1077 reduced total cholesterol by 13.87% in hyperlipidemic patients, and synbiotic foods containing *L. sporogenes* and inulin improved triglycerides and HDL-C (69). Overall, both fermented milk products and probiotics significantly reduced total cholesterol and LDL-C, with interventions exceeding four weeks proving more effective (70).” We revised this paragraph to be more concise while retaining the details and included specific percentages for total cholesterol and triglyceride reductions to facilitate comparison with the cited study. (lines 389-400)
- “These clinical trials have investigated the impact of probiotics on blood pressure, with some reporting modest reductions in SBP reductions (reduced SBP by 3.05 mmHg and DBP by 1.51 mmHg), which is consistent with our work (reduced SBP by 10 mmHg with statistical significance, DBP 5 mmHg with no statistical significance) (75,76). The extent of blood pressure reduction often depends on the baseline levels, the strains of probiotics utilized, and the duration of the intervention.” We revised this paragraph to be more concise while retaining the details and included specific percentages for blood pressure reductions to facilitate comparison with the cited study. (lines 415-417)
- “An increased risk of cardiovascular disease may be closely linked to diabetes mellitus (109). A changed gut microbiome can lead to oxidative stress-related diseases, as well as obesity, metabolic endotoxemia, B-cell dysfunction, and systemic inflammation (110,111). Probiotic therapy may be useful in managing diabetes mellitus because intestinal bacteria produce more SCFAs. Other works demonstrate that probiotics (*B. animalis* subsp. *lactis* BB-12 and *L. acidophilus* La-5) improve glycemic control in patients with type 2 diabetes mellitus, which can reduce FBG and hemoglobin A1c levels by 6.59% and 11.04%, respectively, although these reductions were not statistically significant (112). In support of the current investigation, the FBG level decreased significantly following the use of probiotics compared to the baseline, resulting in reductions of FBG and hemoglobin A1c levels by 0.95% and 3.44%, respectively.” We revised this paragraph to be more concise while retaining the details and included specific percentages for FBG and hemoglobin A1c reductions to facilitate comparison with the cited study. (lines 501-510)

13. Focus discussions on the probiotics that were used. Too much is said about other probiotics not related to the species used.

**Response:** The discussion has been revised to focus primarily on the probiotics used in this study. References to unrelated probiotics have been minimized to maintain relevance.

- “Furthermore, Rabiei et al.’s report revealed a significant decrease in body weight, body mass index, and blood sugar in volunteers with metabolic conditions who received synbiotic products containing seven strains of probiotics, 2x10^8^ CFU/g, along with prebiotics for 12 weeks (29).” This paragraph was removed due to redundancy and lack of relevance to the study. (previous lines 533-537)
- “In this study, the LDL-C levels showed a marked decrease in the probiotic group compared to the placebo group, dropping by 23.37% and 2.94%, respectively, following the intervention. These results are supported by a meta-analysis of randomized controlled trials, which reported that consuming probiotic *Lactobacillus* strains, particularly *L. reuteri* and *L. plantarum*, significantly reduced both total cholesterol and LDL-C levels (69). In a controlled, randomized, double-blind trial conducted among dyslipidemic patients, supplementation with a combination of three *L. plantarum* strains (CECT 7527, CECT 7528, and CECT 7529) resulted in a 17.6% reduction in LDL-C levels after 12 weeks compared to the placebo group (67). In another randomized trial, the administration of *Bifidobacterium longum* CCFM1077 to hyperlipidemic patients reduced LDL-C levels by approximately 13.88%. This benefit may be attributed to the proliferation of anti-obesity-related bacterial genera and favorable shifts in fecal metabolite profiles (77).” We revised the content to make it more concise and incorporated additional studies that closely correlate with our findings to strengthen the discussion and contextualize the results. (line 419-430)
- “In this research, the total cholesterol and triglyceride levels decreased significantly after the consumption of the probiotics for 12 weeks. Compared with pre-intervention placebo values, total cholesterol declined by 16.05% in the probiotic group versus 1.67% in placebo, while triglycerides fell by 7.04% versus 3.54%, respectively. Although HDL-C decreased in the probiotic group, the change was not statistically significant. These results align with previous trials: one involving dyslipidemic patients showed a 13.6% reduction in total cholesterol in the *L. plantarum* group after 12 weeks (67), and another by Keleszade et al. found that six weeks of *L. plantarum* ECGC 13110402 use significantly lowered total cholesterol and LDL-C levels (68). Additionally, *B. longum* CCFM1077 reduced total cholesterol by 13.87% in hyperlipidemic patients, and synbiotic foods containing *L. sporogenes* and inulin improved triglycerides and HDL-C (69). Overall, both fermented milk products and probiotics significantly reduced total cholesterol and LDL-C, with interventions exceeding four weeks proving more effective (70).” We revised the content to make it more concise and incorporated additional studies that closely correlate with our findings to strengthen the discussion and contextualize the results. (line 389-400)

14. In the limitations of the study, indicate possible potential confounders.

**Response:** Potential confounders, such as baseline dietary habits, physical activity, genetic predispositions, and microbiome composition, have been addressed in the limitations section as follows.

- “This study was subject to some limitations, one of which was its use of shotgun sequencing, which produced more information than 16S rRNA V4 region sequencing because of its ability to characterize metabolic pathways and larger gene lists. In addition, potential confounders included baseline variability in diet, physical activity levels, genetic predispositions, and microbiome composition, which may have influenced individual responses to the probiotic intervention. To mitigate adherence-related issues, biweekly follow-ups were conducted during the 12-week intervention to ensure participants achieved at least 80% compliance with the probiotics or placebo regimen. Moreover, despite the lack of standardization in the quality-of-life questionnaire utilized in this trial, the results showed that the intervention was well tolerated and had no negative side effects.” (lines 539-548)

15. Only determine causality in the results obtained when they are absolutely certain that they are exclusively due to the intervention evaluated.

**Response:** Statements regarding causality have been revised to ensure they align with the strength of the evidence presented and do not overstate the findings, as follows.

- Probiotics could influence SBP through several mechanisms related to gut microbiome modulation (71). (lines 401-402)
- “The body's levels of pro- and anti-inflammatory cytokines may be affected by probiotics. Probiotics could improve endothelial function and reduce arterial stiffness, essential for maintaining normal blood pressure, by lowering inflammation, especially in the vascular system (53).” (lines 407-410)
- “The atherosclerotic process may be protected by SCFAs, whereas lipopolysaccharide (LPS) could trigger inflammation and accelerate the onset of atherosclerosis. The proportion of butyric acid-producing bacteria, such as Lachnospiraceae and Ruminococcaceae, declined as CAD progressed, according to research by Liu H. et al. (102).” (lines 478-481)

16. Conclusions are similar to the abstract. Restructure the conclusions.

**Response:** The conclusions have been restructured to provide a more detailed summary of the findings, distinct from the abstract while highlighting the study's implications and future research directions, as follows.

- “In summary, this study showed that *Lacticaseibacillus paracasei* MSMC39-1 and *Bifidobacterium animalis* TA-1 probiotics effectively reduced metabolic syndrome risk factors, including significant decreases in low-density lipoprotein cholesterol, triglycerides, and total cholesterol. Improvements in body weight, body mass index, and waist circumference were observed exclusively in the probiotics group. Gut microbiome analysis revealed enhanced beta-diversity and increased abundance of short-chain fatty acid-producing bacteria (*Blautia, Roseburia, Collinsella*, and *Ruminococcus*), along with predicted functional changes in ribonucleic acid transport, ATP-binding cassette transporters, and lipid metabolism pathways, supporting the probiotics' role in metabolic regulation.” (lines 551-559)

The revisions have greatly enhanced the clarity, accuracy, and overall quality of the manuscript. We are truly grateful for your insightful comments and suggestions, which have strengthened our work. Thank you for considering our revised manuscript for publication.

Once again, we extend our gratitude for your valuable insights and support throughout the peer review process.
